# Beyond innovation: Reimagining inclusive and ethical technologies for ageing populations

**DOI:** 10.1177/20552076261418907

**Published:** 2026-03-16

**Authors:** Claire Gough, Gordana Dermody, Antonio Palmeira

**Affiliations:** 1 64767 College of Nursing and Health Sciences, Caring Futures Institute, Flinders University, Adelaide, Australia; 2 School of Health, 5333 University of the Sunshine Coast, Queensland, Australia; 3 386292 CIDEFES, Universidade Lusofona and CIFI2D Universidade do Porto, Porto, Portugal

**Keywords:** Ageing, innovation, technology adoption, ethics, human factors, care systems

## Abstract

This commentary explores the intersection of technology, ethics and care delivery in the context of ageing populations. It argues that while smart technologies offer promising solutions to support independence and wellbeing, their adoption is shaped by complex human, ecological and philosophical factors. Drawing on theoretical frameworks, such as the bioecological theory and the cyber-ecological model, this article highlights the importance of co-design, cultural sensitivity and ethical integration. Practical examples illustrate how adaptive artificial intelligence, smart homes and other digital tools can enhance care when thoughtfully implemented. The authors call for interdisciplinary collaboration, inclusive design and evidence-based policy to ensure that technological innovation enhances, rather than replaces, human connections in aged care delivery.

As technology continues to evolve, new opportunities are emerging to support older people to age well. Smart technologies have the potential to promote independence, enhance quality of life and enable people to remain in their own homes for longer.^1^ Yet, despite these benefits, uptake among older populations remains limited- often hindered by ageist assumptions and misconceptions about older adults’ interest in or ability to use technology.^2,3^ However, future generations of older people will be increasingly tech-savvy, having grown up with digital devices and online services. This raises a critical question: *How can we ensure that innovation is supported by evidence-based policies and practically integrated into care that empowers older people to engage with and benefit from technology?*

Our aim was to examine the intersection of existing discourses. We argue that this intersection offers a distinct and useful perspective by highlighting commonalities across current frameworks that should be considered as new technologies are developed. Focusing exclusively on a single framework, risks overlooking relevant factors that emerge when different models are considered together. In this sense, a multi-framework approach is needed to adequately support ongoing technological innovation. Such an approach emphasises shared elements across frameworks and leverages the synergies that arise from the interaction between their components.

## Human factors in technology use

It is essential to consider the human factors that influence technology use. These refer to the various aspects, behaviours, capabilities and limitations that influence how people interact with technology.^4^ Psychological, social, practical and contextual factors all play a role in whether technology is adopted and how it is used in both healthcare settings and daily life.^5^ For older adults, technology engagement may be influenced by prior experience, levels of digital literacy and changes in sensory or cognitive function. Personal preferences, confidence and personality traits also matter, as does the attitudes and support of those around them, including healthcare professionals, care workers and family members.

## Challenges in adoption for older people

Older adults face a range of challenges in adopting new technologies, many of which are linked to ethical concerns around consent, privacy, autonomy and dignity. Historical and cultural influences also play a role; for example, early science fiction films often portrayed robots as threatening which may contribute to older adults’ hesitation toward robotic devices.^6^ Therapeutic technologies such as robotic pets have been found to provide opportunities for engagement and reduce social isolation.^7^ However, they can also be perceived as infantising or patronising, especially when they resemble toys, rather than tools to support ongoing wellbeing.^8^

In addition, digital technologies ‒ particularly those that rely on internet connectivity ‒ introduce cybersecurity risks. Concerns about scams, identity theft and data breaches can erode trust in digital tools and services. These fears, combined with a strong preference for face-to-face interactions, contribute to the perception that personal contact is essential to high-quality healthcare and in-home aged care support. As a result, the transition to automated or technology-supported care must be approached with sensitivity to the emotional, social and ethical dimensions that shape older adults’ willingness and ability to engage with technology.

## Challenges in adoption for care providers

Care providers face a range of challenges when it comes to adopting technology in their practice. Concerns around privacy, professional liability and scope of practice can create hesitation among healthcare professionals, particularly when technologies involve data collection, monitoring, or support decision-making. Time constraints and increased workload including responsibilities such as device setup, troubleshooting and maintenance can further discourage adoption, especially in already stretched workforces. In addition, there is a lingering distrust or fear among some care workers that automated technologies could eventually replace human roles, leading to resistance toward technological integration.^9^ These factors highlight the importance of involving care providers in technology design and implementation processes to ensure solutions are practical, ethical and aligned with the realities of day-to-day care.

## Technology acceptance in ageing populations

Understanding how and why older adults adopt or reject technology is critical to integration in aged care settings. The Technology Acceptance Model (TAM) offers a useful framework, suggesting that acceptance largely depends on an individual's perception of a technology's usefulness and ease of use.^10^ Addressing the challenges previously discussed, through thoughtful design, education and inclusive implementation strategies are essential to foster trust and acceptance for carers, older people and their family members.

Practical example

Family members may hesitate to install an in-home monitoring system; their acceptance often hinges on how useful and easy to use they perceive the technology to be.

If they clearly understand how the system can enhance safety, such as detecting falls or sending alerts in emergencies, they are more likely to view it as beneficial (perceived usefulness).

If the system is simple to install, user-friendly and allows control over privacy settings, it reduces barriers to adoption (perceived ease of use).

To support technology acceptance, involving families in the selection process and providing clear demonstrations can improve confidence in the technology, helping to address concerns and support integration into daily routines.

## Philosophy of technology

The *philosophy of technology* is not commonly discussed but relevant in this context as it explores the nature, development and impact of technology on human life, society and knowledge. The *philosophy of technology* examines how technological tools and systems shape our understanding of the world, influence human behaviour and values and intersect with ethical, cultural and political dimensions.^11^ Rather than treating technology as neutral or merely functional, this field emphasises its entwinement with human intentions. It raises critical questions about autonomy, agency and the future, encouraging reflection not only on what technology does, but also on what it signifies and the kind of world it helps to create.

Practical example

A health smart home system is installed to support an older person living alone. Motion sensors and voice-activated assistants help monitor daily routines and detect emergencies, offering peace of mind to both the person and their family.

While this enhances safety and independence (perceived usefulness), it also introduces constant surveillance, which may feel intrusive over time. The older person may begin to question their privacy or feel less in control of their environment, especially if family members or care providers have remote access to the system.

This scenario raises important philosophical questions: Who decides what data is collected and who sees it? And- How does the presence of technology affect the person's sense of autonomy and the nature of care they receive?

Thoughtful integration of smart technologies requires balancing functionality with respect for personal dignity and human connection.

## Ecological view

An *ecological perspective* is both essential and complementary to human factors and individual technology adoption models, such as readiness and acceptance. This is because individuals exist within complex, interrelated systems that shape and constrain their behaviours, decisions and opportunities.^12,13^ Bronfenbrenner's *bioecological theory* emphasises the reciprocal interaction between individuals and their environments, highlighting that human development is not only influenced by personal characteristics but also by dynamic, multi-level systems, ranging from immediate relationships to sociocultural and policy contexts.^14^

Practical example

Whilst an older person may be influenced by a family caregiver or clinician to have a health smart home, they may decide not to adopt it because they are not able to afford it.

Building on this theory, Navarro and Tudge (2022) introduced the concept of *virtual microsystems*- digital environments such as the health smart home sensors that co-exist with physical settings and are embedded within the microsystem layer (e.g., the persons home).^15^ These virtual microsystems enable asynchronous, remote health monitoring and represent a significant evolution in how ecological systems are conceptualised.

**Figure 1.**
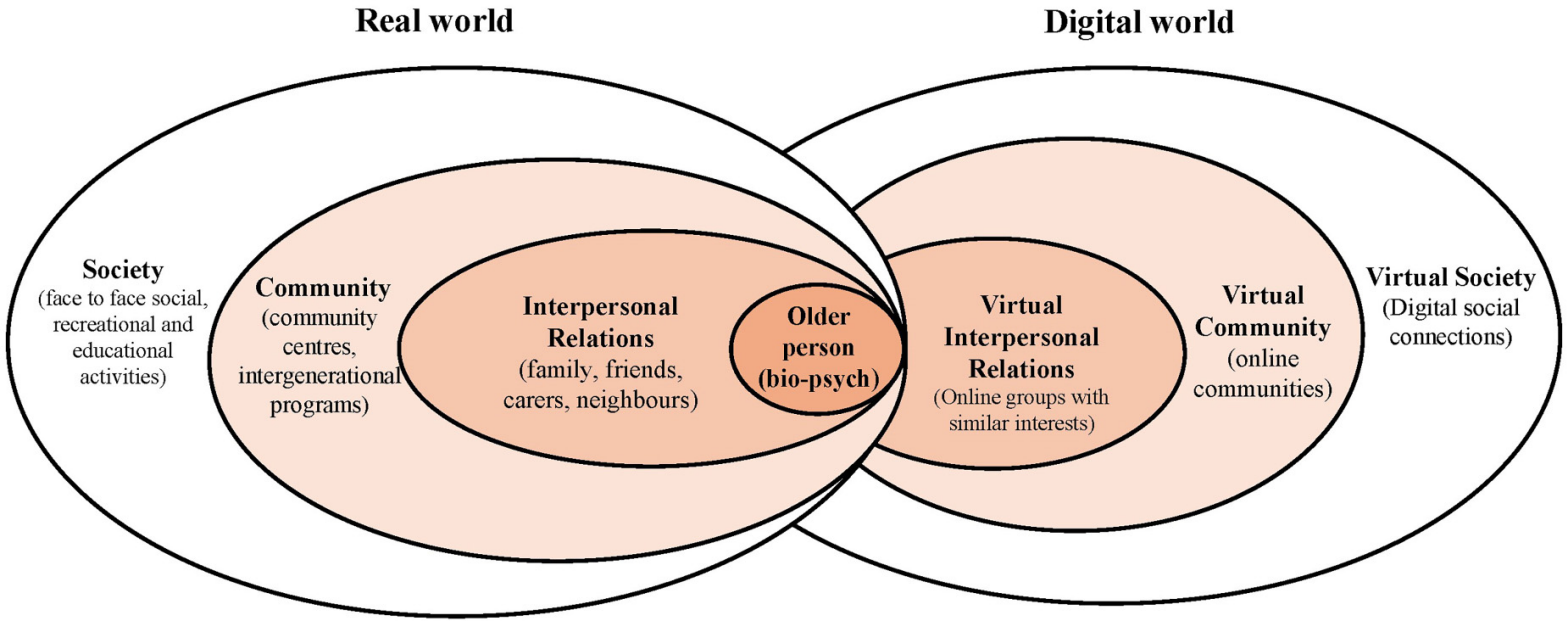
Cyber-ecological approach to understanding the interaction between real and digital worlds for older people.^16^

This shift has previously been discussed in the cyber-ecological model^16^ which proposes a dual layer ecological framework: one grounded in the physical world and the other in the digital realm. In this model, digital agents, such as apps, games or smart technologies exert varying degrees of influence on behaviour, much like the real-world agents (e.g., family, clinicians). Figure 1 provides a visual adaptation of the model that demonstrates the interaction between real and digital worlds for older people.

However, unlike the often top-down and circumstantial influences of the physical world, digital environments are typically entered voluntarily, offering users greater agency and immediate interaction. For example, active video games can promote physical activity as an emergent behaviour of gameplay, demonstrating how digital agents can positively shape health behaviours in ways that are engaging and user driven.^17^

**Figure 2.**
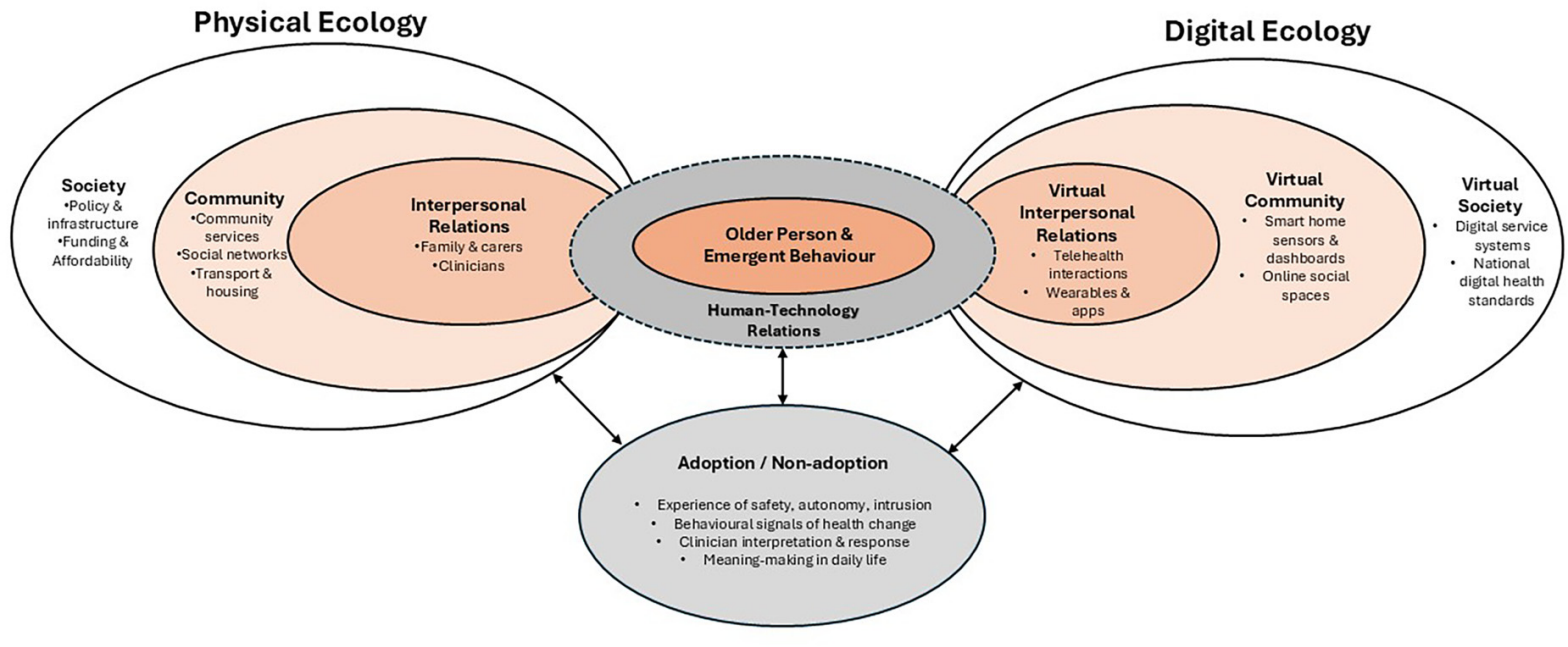
Integrated cyber-ecological and postphenomenological model of human-technology relations in smart-home ageing (adapted from Ref.^16^).

While ecological models emphasise how behaviour is shaped by multi-layered physical and digital systems, postphenomenology complements this by explaining how older adults experience and interpret these technologies through embodied and hermeneutic relations Together, these perspectives allow us to understand both the structural and experiential dimensions of smart-home ageing and technology adoption (presented in Figure 2).

Practical example

An older adult uses a health smart home system that includes wearable devices like a Fitbit, ambient sensors for fall detection and a tablet-based app featuring a virtual caregiver avatar. The app gamifies daily health tasks, such as medication tracking and step goals and provides real-time feedback and encouragement, influencing their health behaviour through ongoing digital interaction.

## Future directions in ageing and technology

To integrate emerging technologies effectively and embed them in a person's home, we need stronger emphasis on implementation science to bridge the gap between innovation and real-world application. Policy support is vital, providing evidence-based frameworks and funding to encourage adoption. Equally important is the co-design of technologies with the people that will use them, ensuring that solutions are tailored to the needs and preferences of older people.

## Human factors and changing demographics

As older populations become more tech-savvy, traditional barriers are diminishing. However, human factors, especially caregiver and family attitudes, remain critical. Education and training for healthcare professionals can improve digital literacy and reduce resistance, fostering a more inclusive environment for technology in aging care.

## Artificial intelligence

In the years to come, there will be increasing efforts to personalise technology for older adults, focusing on learning how to better engage with technology despite the challenges that aging presents. The growing use of artificial intelligence (AI) aims to enhance user interfaces and deliver experiences specifically designed for the user, in this case older people.^18^ This process includes the development of culturally sensitive AI policies, practices and products that address the distinct needs of aging populations.^19^ Adaptive AI mechanisms are currently being designed to offer enjoyable and meaningful content for older users.^20^ For instance, virtual reality combined with AI-powered games such as fishing simulations has demonstrated effectiveness in improving reaction time and attention, while tailoring content to individual performance.^20^ However, future use of AI to support ageing will depend on overcoming current limitations (e.g., underrepresentation of older people in data sets used to train AI) and harnessing AI's transformative potential to support healthy aging across areas such as nutrition and physical activity.

Practical example

An older adult uses a culturally adapted AI-powered nutrition app that tracks meals via voice input and provides personalised dietary suggestions. The app adjusts its interface to accommodate slower speech and reduced dexterity and uses adaptive learning to tailor recommendations based on the user's preferences and health goals.

## Research and funding trends

Europe is shifting toward Investigator-Initiated Studies (IIS), allowing researchers to define their own questions. This bottom-up model encourages collaboration across gerontology, gerontechnology, human computer interaction and implementation science. Such funding streams now explicitly welcome projects on smart-home monitoring, robotics and ethical AI, accelerating real-world pilots and pragmatic evaluation.^21^ By giving researchers control of study design and intellectual property, these programs shorten the path from concept to real-world pilots and make it easier to embed pragmatic evaluation in diverse care settings, from rural community clinics to urban assisted-living complexes.

Parallel advances in miniaturised biosensors and edge-AI are enabling continuous capture of gait, sleep, meal composition and medication adherence data; early EU-funded trials already feed these multimodal streams into adaptive algorithms that generate personalised coaching or trigger just-in-time interventions.^21^ Over the next decade, we anticipate that diet-responsive micro-capsules and implantable nanorobots ‒ capable of releasing nutrients, insulin or anti-inflammatory agents ‒ will be coupled with cloud-based decision engines. So that the same platform can shift seamlessly between prevention and treatment modes. Achieving this vision will require strict data-governance frameworks and new interdisciplinary IIS protocols, but the payoff is a learning health-ecosystem in which every older adult's daily behaviour continuously refine both their individual care plan and population-level models of healthy ageing. Additional funding agencies may subsequently offer grant opportunities akin to those currently available within the European Union.

## Policy implications for ethical technology integration

Dedicated and clearly defined funding streams are needed to explicitly support ethical technology adoption across the aged and community care sectors, including investment in implementation science, workforce capability building and ongoing system integration. To ensure ethical and inclusive adoption of technology to support ageing populations, national guidelines must be co-designed with older adults, family, caregivers, community nurses, the home health care industry and interdisciplinary experts to reflect lived experience and diverse care needs. Policies should include older adults in AI datasets to prevent bias and enhance relevance to support quality outcomes. Robust standards for data governance, privacy and informed consent must underpin all technology devices safeguarding autonomy and trust for older populations and their families. Finally, equity must be central to policies, addressing regional and rural disparities in infrastructure and digital literacy, ensuing that older people in all communities can access and benefit from technology innovations.

## Nursing implications

To evolve and keep pace with ethical digital health integration nursing curricula should embed content on digital ethics, data governance and patient autonomy to prepare nurses for technology enabled care. Ongoing support will be needed to support nurses to interpret digital data within clinical contexts and make informed patient-centred decision.^22^ Training programs should prioritise co-design and digital literacy, enabling nurses to direct technologies to meet a need and support efficient and safe care delivery. As AI becomes integral to assessment and care planning, nurses should be well equipped to critically appraise output and integrate AI insights into care (Table 1).

## Discussion

This commentary integrates ethical, ecological and experiential perspectives to explain the complexity of technology adoption for use with ageing populations, and crucially, what nursing and policy must do next. Ecologically, adoption is shaped by multiple influences from interpersonal relationships to community policy, whilst postphenomenology clarifies how older adults experience technologies as empowering, intrusive or ambiguous in daily life. Considered together (and visualised in Figures 1 and 2), these perspectives explain why promising pilot studies seldom scale up without workforce capability, procurement, funding and governance affecting uptake.^1,12–16^ They also highlight the influence of human factors, that often persist despite increased technology competence ‒ such as trust, dignity and relational care ‒ all factors that nurses are uniquely positioned to deliver.

Within the cyber-ecological model, nurses operate across physical and digital microsystems (such as home care and telehealth) translating data into person-centred decisions while maintaining relational safety. Evidence shows that acceptance hinges on perceived usefulness and ease of use, but adoption falters when clinical interpretation is weak or role demands and burden rises; nursing roles in triaging alerts, and calibrating consent therefore become decisive.^10,22^ Postphenomenological insights reinforce this by placing the lived experience at the forefront, where nurses mitigate risk of infantilisation through co-design and reframe devices as tools for autonomy rather than replacements for face-to-face human contact.^6–8^^,11^ In practical terms, this means embedding digital ethics, data governance and AI appraisal into nursing curricula, building capacity to analyse and interpret sensor data, and establish protocols for shared decision-making where older adults and family caregivers can opt in or out of features.^9,10,22^

At the macrosystem level, targeted funding and standards must align with ecological realities of affordability, infrastructure and workforce time. Without policy that covers device procurement, maintenance and implementation support, microsystem readiness will continue to act as a barrier to adoption- even when older people and staff are supportive.^12–15^^,21^ Policies mandating co-design and transparency are not only ethically sound, but they also have the potential reduce resistance by clarifying data flows and consent, both key concerns for older adults and care providers.^3,8,11^ Equity provisions are therefore essential, as rural and lower literacy populations remain underrepresented in AI training sets and underserved by infrastructure, inclusion standards and capability-building funds are necessary to avoid and prevent algorithmic bias.^18,19,21^ Policy should recognise nursing as a digital health linchpin, resourcing protected time for digital assessment, interpretation, care planning and specific governance frameworks should ensure that automated activities do not displace clinical judgement.^9,21,22^

The evidence demonstrates that smart-home technologies can improve quality of life, but these gains often depend on nursing-led interpretation and service integration, without this, alerts become noise and trust in technology fades.^1,22^ Challenges with care robots and AI highlight the need for co-design, dignity-preserving aesthetics and clear role boundaries, alongside pragmatic policies for dataset inclusion and auditability to prevent bias.^6–8^ TAMs remain useful yet are insufficient. Implementation research must address workflow, liability and explainability to support nurses to delivery safe, quality care.^10^

To operationalise these insights into care delivery, priorities include embedding digital ethics and AI critique into nursing education, scaling co-design with older adults and caregivers, aligning policy with funding and human oversight and ensuring equity through inclusive datasets and infrastructure investments. These actions link ecological and ethical frameworks with nursing and policy, enabling technology to enhance ‒ not replace ‒ human care.

## Conclusion

The future of ageing extends beyond technological innovation- it is a human and systems challenge that requires thoughtful integration of care, community and context. As we stand at the intersection of innovation, ethics and practical care, we must ensure that emerging technologies are not only functional but also meaningful, respectful and inclusive. This requires more than just devices; it demands a shift in mindset, towards codesign with older adults, investment in implementation science and policies that prioritise dignity, autonomy and equity. For nursing, this means embedding ethical digital health content into education, giving nurses the skills to interpret data in context and prepare them for AI-enabled assessment and care planning. Nurses will play a pivotal role in ensuring that technology enhances ‒ not replaces ‒ human connection by advocating for transparency, cultural safety and relational care. By aligning human factors, ecological systems and philosophical reflection with practical innovation, we can build a care ecosystem that learns, adapts and empowers every older person to age well, supported by skilled professionals who integrate technology with compassion.

**Table 1.** Practical strategies for ethical technology integration.

Ethical principle	Practical ‘How to’
Respect autonomy and informed preference	Provide clear, jargon free explanations of technology options. Give opportunities for free choice using opt-in/opt-out features. Provide opportunities for co-design/co-creation.
Preserve dignity and avoid infantilisation	Use age- appropriate designs (avoiding toy-like appearances) that support framing technology as empowering.
Prioritise cultural safety	Incorporate cultural norms and language preferences in design by engaging cultural representation in early design phases.
Consider relational impact	Assess how technology affects family and family caregiver dynamics by including relational questions in evaluation tools and feedback opportunities.
Provide human oversight	Ensure that humans review automated alerts and decisions. Retain human involvement to ensure safe care delivery. Involve community nurses and home care provider in co-design/co-creation.
Ensure transparency and explainability	Use plain language to explain how data is collected, stored and used, and privacy and data protection.
Engage users in co-design	Involve older adults, community nurses and family caregivers in technology design and testing from inception- ensuring older adults are involved in testing and feedback phases.
